# Goats distinguish between positive and negative emotion-linked vocalisations

**DOI:** 10.1186/s12983-019-0323-z

**Published:** 2019-07-10

**Authors:** Luigi Baciadonna, Elodie F. Briefer, Livio Favaro, Alan G. McElligott

**Affiliations:** 1 Biological and Experimental Psychology, School of Biological and Chemical Sciences, Queen Mary University of London, Mile End Road, London, E1 4NS UK; 2 Institute of Agricultural Sciences, ETH Zürich, Universitätsstrasse 2, 8092 Zürich, Switzerland; 3 Department of Life Sciences and Systems Biology, University of Turin, Via Accademia Albertina 13, 10123 Turin, Italy; 4 Centre for Research in Ecology, Evolution and Behaviour, Department of Life Sciences, University of Roehampton, London, SW15 4JD UK; 5 Behavioural Ecology Group, Section for Ecology & Evolution, Department of Biology, University of Copenhagen, 2100 Copenhagen Ø, Denmark; 6 Equipe Neuro-Ethologie Sensorielle, ENES/Neuro-PSI CNRS UMR9197, University of Lyon/Saint-Etienne, Saint-Etienne, France

**Keywords:** Bioacoustics, Emotions, Heart-rate variability, Playback, Positive and negative valence, Ungulates

## Abstract

**Background:**

Evidence from humans suggests that the expression of emotions can regulate social interactions and promote coordination within a group. Despite its evolutionary importance, social communication of emotions in non-human animals is still not well understood. Here, we combine behavioural and physiological measures, to determine if animals can distinguish between vocalisations linked to different emotional valences (positive and negative). Using a playback paradigm, goats were habituated to listen to a conspecific call associated with positive or negative valence (habituation phase) and were subsequently exposed to a variant of the same call type (contact call) associated with the opposite valence (dishabituation phase), followed by a final call randomly selected from the habituation phase as control (rehabituation phase). The effects of the calls on the occurrence of looking and cardiac responses in these phases were recorded and compared.

**Results:**

We found that when the valence of the call variant changed, goats were more likely to look at the source of the sound, indicating that they could distinguish calls based on their valence. Heart rate was not affected by the valence of the calls played, whereas heart-rate variability tended to be higher in the habituation and rehabituation phases, when positive calls were played compared to negative ones. Together, the behavioural and physiological measures provide evidence suggesting, first, that goats are able to distinguish call variants based on their valence, and second, that goat behaviour and cardiac responses are affected by call valence.

**Conclusion:**

This study indicates that auditory modalities are a potent means to communicate emotions in non-human animals. These findings can contribute to our understanding of the evolution of emotion perception in non-human animals.

## Background

Emotions have an adaptive value because they allow animals to respond appropriately to salient stimuli. Negative emotions enable individuals to respond appropriately to potentially life-threatening situations. Positive emotions, by contrast, guide responses to stimuli or events that enhance fitness and widen the individual cognitive and behavioural repertoire [1–4]. Given the adaptive importance of emotions, their occurrence should be phylogenetically widespread, and their basic underlying mechanisms might be preserved across taxa [5].

In comparative psychology, substantial progress has been made in identifying animal emotions using behavioural [6, 7], physiological [8], and cognitive indicators [9, 10]. Accordingly, emotions are often accompanied by visible changes in a subject’s facial expression, behaviour [11, 12] and vocalisations [6, 13, 14]. Although emotion-related changes are not necessarily intentionally communicated, they could be used as cues to the emotional states of conspecifics [15, 16]. Behavioural and physiological responses of the receivers of the cue can be used to assess whether these animals simply perceive the difference between emotional stimuli or whether they are also affected by these stimuli in a way that matches the emotion of the producer of the cues [17–21].

Non-human animals are able to perceive the emotional state of conspecifics and even heterospecifics (including humans) by using one sensory modality or a combination of sensory modalities [22–24]. Additionally, state-matching of emotions between producers and receivers has been shown in some species [20, 25, 26]. For example, exposure to odour or vocal cues from a stressed individual in cattle (*Bos taurus*) and pigs (*Sus scrofa*) or witnessing a family member being involved in an agonistic interaction in geese (*Anser anser*) can affect the behaviour of the subject by increasing fearfulness and modifying physiology (cortisol level and heart rate; 25–27) [26–29]. Horses (*Equus caballus*) show a left gaze bias and increased heart rate when facing photos of angry human faces compared to happy human faces [30]. In rodents, exposure to negative ultrasonic calls causes anxiety-related behaviours, while exposure to positive sounds triggers approach behaviours [31, 32]. Further evidence is required to understand the mechanism through which emotions affect conspecifics [33].

Goats (*Capra hircus*) are highly social and are an excellent model to investigate the mechanisms underlying the social dimension of emotions. Goat contact calls encode important information about the arousal and valence of the emotional state of the caller, along with information on caller’s individuality, sex and age [6, 30]. Accordingly, it is likely that the expression of emotions in goat contact calls can be detected by other members of the group in a similar way to other types of information [33–35]. In addition, goats are sensitive to human facial expressions and show a preference for happy compared with angry faces [36]. The aims of this study were to investigate whether goats can discriminate conspecific calls conveying positive and negative emotional information and to assess the potential impact of the emotional valence conveyed by the calls on the behavioural and physiological responses of receivers. In particular, to achieve our aims, we used a habituation-dishabituation-rehabituation playback design [37], in which conspecific contact calls recorded during situations triggering emotions of positive or negative valence were played back to the goats in the habituation phase, before changing the valence in the dishabituation phase. We expected to find an increased level of attention (looking towards the source of the sound for longer) when the dishabituation stimulus was played, if perceived as different from the calls used in the habituation phase. In addition, to verify that the response pattern in the dishabituation phase was not due to a random change of attention, the playback ended with the same variant of calls used during the habituation phase. In this control condition, we expected shorter duration of looking towards the source of the sound compared to the dishabituation phase, because the calls would have been played already during habituation.

We assessed the effect of the perception of emotional-linked calls at a physiological level by recording heart rate (HR) and heart-rate variability (HRV) during the playback experiments. HR is controlled by activation of the sympathetic (increase in HR) and vagal (decrease in HR) systems and therefore is considered an indicator of emotional arousal [7, 38]. By contrast, HRV is mainly under the vagal regulation and thus indicates when only the vagal branch of the autonomic nervous system is activated [39, 40]. Because low HRV is associated, in humans, with depression and post-traumatic stress disorder, it has been proposed as a potential indicator of emotional valence in other species [39–43]. Overall, physiological parameters can provide strong evidence in addition to behavioural data when investigating the arousal and valence of emotions [7, 44, 45]. We predicted that HR would decrease during the habituation phase and increase when the dishabituation stimulus was played regardless of the valence of calls, while the opposite would occur for HRV. We hypothesized that HRV, because it negatively correlates with HR, would increase over time during the habituation phase. Furthermore, in the dishabituation phase, we expected an increase in HR and therefore predicted reduced HRV regardless of call valence upon hearing the first call. We also expected an increase in HRV over the two subsequent calls, as for the habituation phase.

In this study, we considered several critical issues often not controlled in related research: 1) the emotional state of both the caller and the receiver were assessed, and 2) only contact calls were used, so that the reaction of the receiver would be purely dependent on the encoded emotions rather than the type of vocalisations [46].

## Results

### Occurrence of looking towards the speaker

**Fig. 1:**
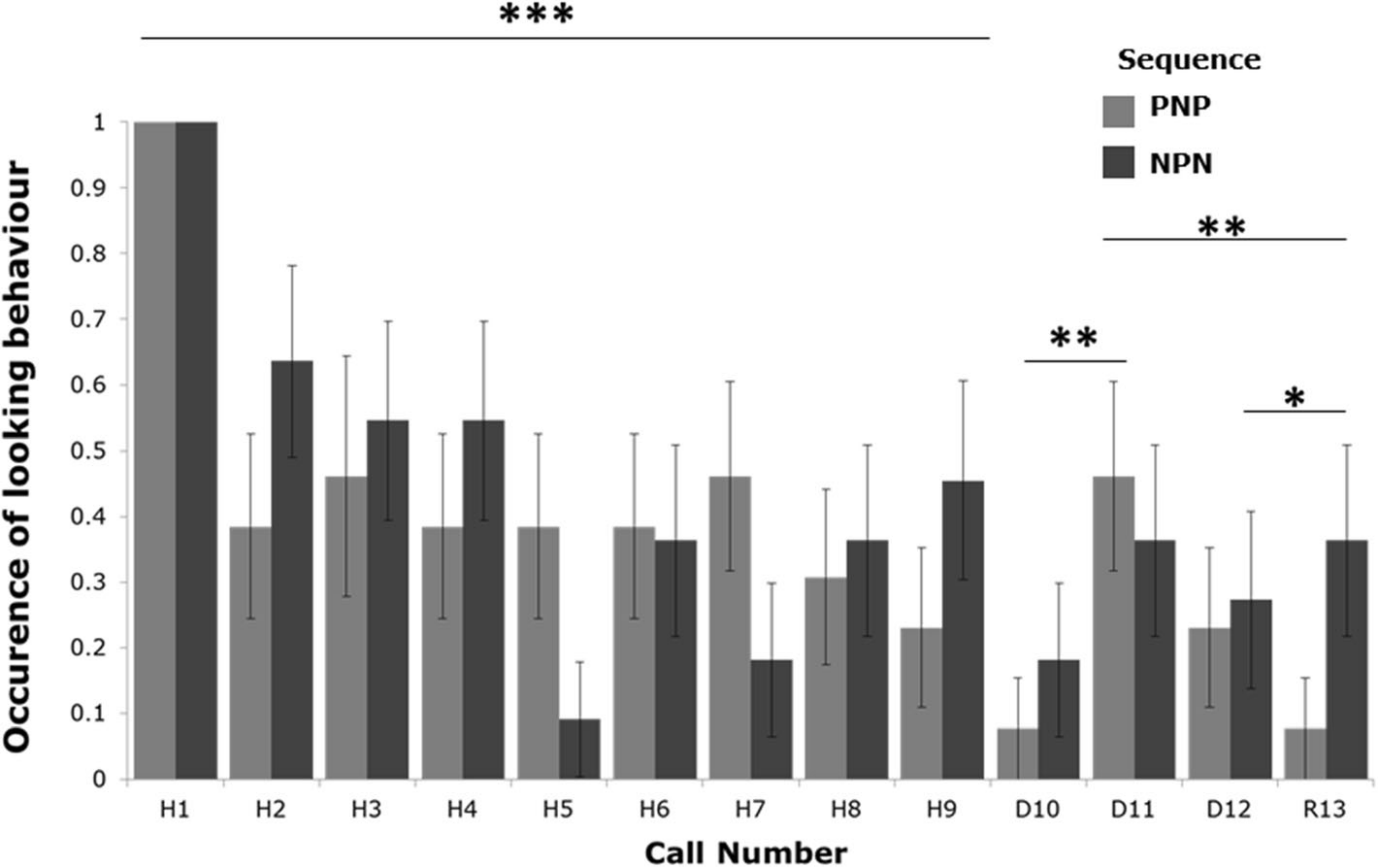
Occurrence of looking in response to the playbacks. The mean +/− SE occurrence of looking towards the loudspeaker is indicated in light grey for Positive (H1-H9)-Negative (D10-D12)-Positive (R13) call sequences in dark grey for the Negative (H1-H9)-Positive (D10-D12)-Negative (R13) call sequences. *** *p* < 0.001; ** *p* < 0.01; * *p* < 0.05

During the habituation phase (calls H1-H9), goats reduced the occurrence of looking towards the speaker (Generalised Linear Mixed-Effect Model: *χ*^2^_(1)_ = 30.01, *p* < 0.0001; Fig. 1), indicating that they habituated to the call type, regardless of call valence (GLMM; valence: *χ*^2^_(1)_ = 0.13, *p* = 0.71; interaction between call number and valence: *χ*^2^_(1)_ = 0.26, *p* = 0.60). There was a tendency for goats to further reduce the occurrence of looking between the last call of habituation (H9) and the 1st call of dishabituation (D10; GLMM; *χ*^2^_(1)_ = 3.76, *p* = 0.052), regardless of the valence of the calls (GLMM; valence: *χ*^2^_(1)_ = 0.18, *p* = 0.66; interaction between call number and valence: *χ*^2^_(1)_ = 1.63, *p* = 0.20). When the last call of habituation (H9) and the 2nd call of dishabituation (D11) were compared, we did not find any significant effect of call number, valence or their interaction (*p* ≥ 0.18). During the dishabituation phase, subjects increased the occurrence of looking between the 1st (D10) and the 2nd call (D11; GLMM; *χ*^2^_(1)_ = 5.58, *p* = 0.018), regardless of the valence of the calls (GLMM; valence: *χ*^2^_(1)_ = 0.004, *p* = 0.94; interaction between call number and valence: *χ*^2^_(1)_ = 0.88, *p* = 0.34). Call number, valence or their interaction (*p* ≥ 0.53) were not significant when the 2nd (D11) and 3rd (D12) calls were compared. Finally, the occurrence of looking decreased between the 2nd call of dishabituation (D11) and the rehabituation call (R13; G LMM; χ^2^_(1)_ = 8.12, *p* = 0.004). Additionally, they looked more at the call (D11 or R13) that was negative (frustration and isolation calls combined; mean ± SD = 0.41 ± 0.14) compared to the positive call (0.21 ± 0.11; G LMM; *χ*^2^_(1)_ = 8.12, *p* = 0.004). The interaction between call number and valence was not significant (D11 vs R13; *χ*^2^_(1)_ = 0.00, *p* = 1.00). Call number, valence or their interaction on the occurrence of looking between the 1st dishabituation call (D10) and the rehabituation call (R13; *p* ≥ 0.40) were not significant. When the 3rd call of dishabituation (D12) and the rehabituation call (R13) were analysed, we found that goats looked more at the call (D12 or R13) that was negative (mean ± SD = 0.29 ± 0.13) compared to the positive call (0.17 ± 0.10;GLMM; *χ*^2^_(1)_ = 5.38, *p* = 0.020). However, call number (*χ*^2^_(1)_ = 1.68, *p* = 0.19) and the interaction between call number (D12, R13) and valence (*χ*^2^_(1)_ = 0.20, *p* = 0.65) were not significant. In summary, when the valence changed from the last call of habituation to the first call of dishabituation, goats tended to decrease the occurrence of looking. This behaviour then increased to reach a similar value as at the end of the habituation when the second call of the dishabituation was played back. This suggests an ability to detect a change in call valence, indicated by a drop in the occurrence of looking suggesting a freezing response.

### Physiology: heart rate (HR) and heart rate variability (HRV)

**Fig. 2:**
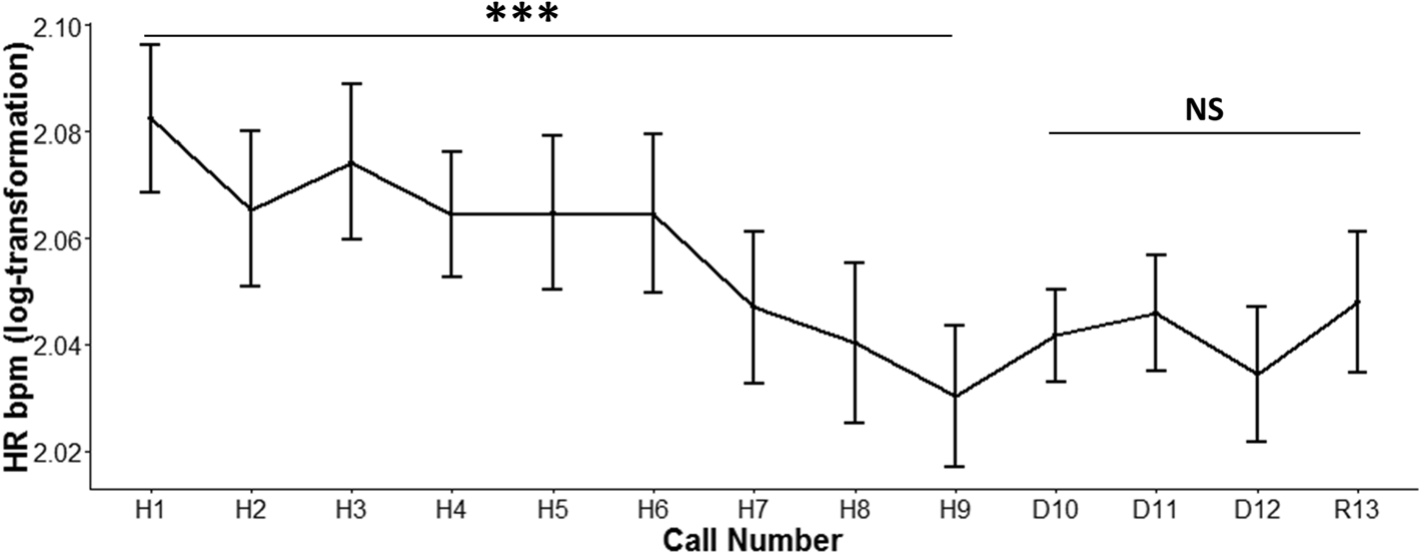
Heart rate during the playbacks. Mean +/− SE heart rate (HR) during the habituation phase (H1-H9), dishabituation phase (D10-D12) and rehabituation phase (R13). During the habituation phase, HR decreased and did not vary significantly throughout dishabituation and rehabituation. *** *p* < 0.001; NS = not significant

HR decreased during habituation (calls H1-H9; LMM; *χ*^2^_(1)_ = 26.24, *p* < 0.001; Fig. 2), but did not change during the dishabituation or rehabituation phases. Neither the valence of the calls played during the habituation phase (LMM; *χ*^2^_(1)_ = 2.50, *p* = 0.11) nor the interaction between call number (H1-H9) and valence (LMM; *χ*^2^_(1)_ = 0.31, *p* = 0.57) had an effect on HR. When the last habituations call (H9) and the dishabituation calls (D10, D11, and D12) were analysed, HR was not affected by call number, valence or their interaction (*p* ≥ 0.97). When the dishabituation calls (D10 vs D11 and 11 vs 12) were considered, HR was not affected by call number, valence or their interaction (*p* ≥ 0.09). Finally, when the calls of dishabituation (D10, D11, and D12) and the rehabituation call (R13) were considered, HR was not affected by call number, valence or their interaction (*p* ≥ 0.23).

**Fig. 3:**
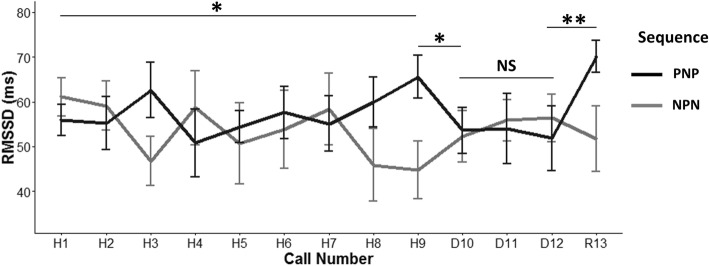
Heart-rate variability (RMSSD) in response to the playbacks. Mean +/− SE RMSSD during the habituation phase (H1-H9), dishabituation phase (D10-D12) and rehabituation phase (R13). The black line (PNP) represents the sequence positive (habituation) – negative (dishabituation) – positive (rehabituation) calls and the grey line (NPN) represents the sequence negative (habituation) – positive (dishabituation) – negative (rehabituation) calls. The habituation phase revealed an interaction effect between the valence of the call broadcasted and the call number (H1-H9). The comparison between the last call of habituation (H9) and the 1st call of dishabituation (D10) revealed an effect of valence. An interaction effect between call number and valence was found when the 3rd call of dishabituation (D12) was compared with the rehabituation call (R13). *** *p* < 0.001; ** *p* < 0.01; * *p* = 0.052; NS = not significant

There was a possible interaction effect between valence and call number for heart-rate variability during habituation (calls H1-H9; LMM; *χ*^2^_(1)_ = 3.75, *p* = 0.052; mean ± SD positive valence = 57.16 ± 1.87 ms vs negative valence = 53.55 ± 2.39 ms; Fig. 3). However, post-hoc Tukey tests investigating the valence effect on each habituation call did not reveal any statistical differences in HRV between positive and negative calls (*p* > 0.74). The comparison between the last call of habituation (H9) and the 1st call of dishabituation (D10) revealed that HRV was higher for the call (H9 or D10) that was positive (mean ± SD = 59.59 ± 4.95 ms) compared to the negative call (48.53 ± 6.1 ms; LMM; *χ*^2^_(1)_ = 4.37, *p* = 0.036), regardless of call number (LMM; *χ*^2^_(1)_ = 0.03, *p* = 0.86; interaction between call number and valence LMM; *χ*^2^_(1)_ = 1.58, *p* = 0.20). The comparison between the 1st (D10) and 2nd (D11), and between the 2nd (D11) and 3rd (D12) calls of dishabituation did not reveal any significant effect of call number, valence or their interaction (*p* ≥ 0.61). Finally, the 3rd call (D12) of dishabituation was compared to the rehabituation call (R13) and an interaction effect was found between call number and valence (LMM; *χ*^2^_(1)_ = 4.36, *p* = 0.036). Post-hoc analyses revealed a tendency for the HRV to be lower for negative rehabituation calls (R13; mean ± SD = 51.76 ± 7.33 ms) than for positive rehabituation calls (70.12 ± 3.52 ms; Tukey HSD; *z* = 2.45, *p* = 0.064). There was also a tendency for the HRV to be higher when the rehabituation call was positive (R13; mean ± SD = 70.12 ± 3.52 ms) than when the 3rd dishabituation call was positive (D12; 51.83 ± 7.20 ms; *z* = 2.44, *p* = 0.067). All the other comparisons included in the post-hoc analyses were not significant (*p ≥* 0.17). In summary, HR decreased during habituation and did not change in the dishabituation and rehabituation phases. Heart-rate variability tended to be higher when positive calls were played compared to negative ones in the habituation and rehabituation phases.

## Discussion

The ability of goats to discriminate the emotional valence conveyed by conspecific calls and the effect of these calls on their physiology were investigated using a habituation-dishabituation-rehabituation paradigm. We provide evidence which suggests that goats are probably able to discriminate between conspecific calls associated with opposing emotional valences. Behavioural and physiological responses suggest that the discrimination occurred with a short delay and on the second call of dishabituation. Our study suggests that vocalisations are a potentially powerful channel for expressing emotions that can be perceived by conspecifics. It therefore paves the way for the investigation of the evolutionary importance of emotional perception in non-human animals [19, 25, 26].

As expected, during the habituation phase, goats gradually reduced the duration of looking towards the sound source, and heart rate also decreased, suggesting that habituation to the valence of the stimuli occurred. Later, the occurrence of looking increased immediately after the second call of dishabituation, and decreased during rehabituation, suggesting that goats perceived the change in call valence. Since all calls played back were of the same call type, this suggests the ability of goats to perceive the calls as different based solely on valence information [47–49]. The calls played during the dishabituation phase differed in valence compared to those played in the habituation phase, but were not different in amplitude (loudness), because the stimuli had been rescaled to the same maximum amplitude or onset. The delay in the behavioural response, in comparison with the faster physiological reaction, could be explained by the way in which acoustic stimuli are processed. Stimuli that are loud and have abrupt onsets are more efficient at inducing responses [50]. These stimuli induce visible changes within a short period of time (10 ms) at behavioural (e.g. stopping ongoing activity, moving the body towards the source of the noise) and physiological levels (increase in heart rate and blood pressure), similarly to those that we would expect to occur when, for example, a first call of habituation is played [50, 51]. This strong reaction is caused by direct circuits connecting the auditory nerve to posterior parts of the brain (i.e. nucleus pontis caudalis of the reticular formation; [50]). In our experiment, by contrast, the calls played during the dishabituation phase differed from those played in the habituation phase only by their context of production. The absence of variation in the heart rate could be due to the fact that the stimuli used had a relatively similar arousal level and, in addition, the call used in each sequence belonged to the same subject. We suggest that the subtle and slower behavioural response could be due to the regulation of the emotional changes in the listener. The regulation of emotional states is controlled by cholinergic and dopaminergic systems [51, 52] and potentially by the amygdala receiving projections from the thalamus, directly connected with the cochlear root neurons [53]. We interpret the short time delay (2 calls over 40 s) in goat responses to the dishabituation calls as resulting from the time needed to process the emotional change conveyed in the call [51–53]. Indeed, the response observed during the first call of dishabituation (low looking duration) might be explained by a “freezing” response, where goats were gathering more information on the valence of the calls before reacting to it [54]. In the rehabituation phase, the occurrence of looking increased when negative calls were played compared with positive calls. In evolutionary terms, it is important to attend to negative signals with life-threatening consequences, and the responses of the goats are in line with this expectation [2, 48, 55, 56].

During the habitation phase, there was a tendency for heart-rate variability to be higher when positive calls were played back compared to negative ones. In addition, heart-rate variability was higher in the rehabituation phase when the calls were positive. Several studies proposed that heart-rate variability is a reliable indicator of emotional valence in non-human animals [42, 43, 57, 58] and humans [59, 60]. However, we suggest that this has to be further investigated, especially when the situations inducing positive and negative emotional states are characterised by different levels of arousal [7, 61, 62]. In the present study, positive calls induced higher heart-rate variability of goats, indicating greater involvement of the parasympathetic over the sympathetic system during the habituation phase [39]. Thus, we suggest that goats were experiencing relatively low levels of arousal throughout the dishabituation and rehabituation phases. The HR was low throughout the phases of the playback and vagal activation was high in response to positively-valenced calls because HRV increased upon hearing positive calls.

Heart rate gradually decreased during the habituation phase and did not increase when the valence of the call changed, both in the dishabituation and rehabituation phases. Heart rate is usually affected by the type of signal presented and the physiological state of the animal [63]. In domestic ungulates, this parameter increases mainly when hearing sudden noises, during novel object presentation and when unpredictable events are presented [64]. Goats experienced a stable level of HR during dishabituation and rehabituation phases, even when the call valence changed. Nevertheless, heart-rate variability, suggests that goats discriminated the subtle information about valence conveyed by the call structure.

To summarise, our combined behavioural and physiological measures provide evidence that non-human animals can discriminate subtle changes within call types as a result of the emotional valence experienced by the producers. Perceiving the emotional state of another individual through its vocalisations and being affected by those vocalisations have a strong adaptive value considering the dynamics of social organisations where, for example, group size and composition changes over time. Many social animals live under environmental conditions where individuals are not always in visual contact with one another during the day or night [65, 66], and therefore, could acquire an evolutionary advantage through the discrimination of the emotional content of conspecifics’ calls [67]. Furthermore, expressing emotions using vocalisations and being able to detect and share the emotional state of a conspecific may facilitate motor coordination among the individuals in a group and strengthen social bonds and group cohesion [21, 22, 68–70].

## Conclusion

Although a substantial amount of research has been done to investigate emotion expression in non-human animals and to reveal their importance for survival at the individual level, the investigation of emotion perception in group living animals is sparse [21]. Here we provide evidence for the ability of non-human animals to discriminate emotions conveyed in calls emitted by conspecifics. We also provide evidence for the impact of emotionally-valenced calls on the behaviour (occurrence of looking) and physiological responses (HRV) of goats. When the behavioural and physiological parameters are combined, our results suggest that non-human animals are not only attentive, but might also be sensitive to the emotional states of other individuals.

## Methods

### Subjects and experimental apparatus

**Fig. 4:**
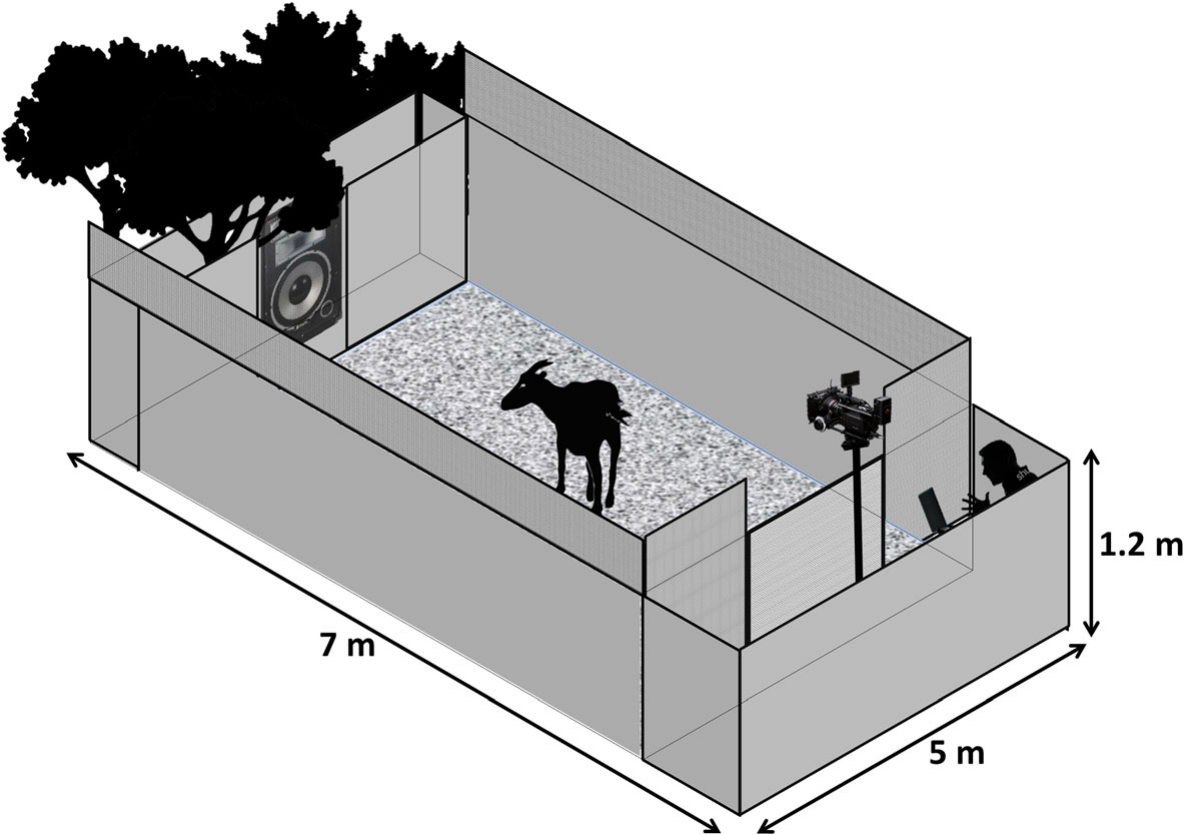
Experimental enclosure. The experimental apparatus (7 m × 5 m) consisted of a start pen connected by a door to a central arena. The loudspeaker was placed at the far end of the arena (outside the perimeter) and was covered with hunting net and natural vegetation. The experimenter remained inside the start pen during the tests, out of view, behind a PVC garden screening fence

The study was carried out at Buttercups Sanctuary for Goats (http://www.buttercups.org.uk) in Kent, UK. At the sanctuary, goats are released into a large field during the day and are confined indoors either in individual or shared pens (average size = 3.5 m^2^) at night. Goats have ad libitum access to hay, grass, and water and are also fed with a commercial concentrate according to their health condition and age. In total, 24 adult goats (12 females and 12 castrated males) of different breeds and ages (Table 1) were tested from May to September 2015, at Buttercups Sanctuary for Goats in Kent (UK). An experimental arena (7 m × 5 m) was set up and placed in one of the fields where the goats are released during the day. The arena consisted of a rectangular area composed of a start pen connected by a gate to a central arena made with a commercial opaque agricultural metal fence (Fig. 4). A loudspeaker was placed outside the perimeter of the arena, on the opposite side to the main gate. The speaker was not visible to the goats and was concealed with camouflage netting.

**Table 1:** Goats tested and experimental design. PNP indicates a Positive (habituation) - Negative (dishabituation) - Positive (rehabituation) sequence; NPN indicates a Negative (habituation) - Positive (dishabituation) – Negative (rehabituation) sequence. FEFR indicates sequences built with FEeding anticipation and feeding FRustration calls; FRFE indicates sequences built with feeding FRustration and FEeding anticipation calls; FEIS indicates sequences built with FEeding anticipation and ISolation calls and ISFE indicates sequences built with ISolation and FEeding anticipation calls

D	Sex	Age	Group	Session	Playback Sex	Sequence
1	Male	8	1	1	Male	PNP (FEFR)
				2	Male	NPN (FRFE)
2	Male	NA	1	1	Male	PNP (FEIS)
				2	Male	NPN (ISFE)
3	Male	7	1	1	Male	PNP (FEFR)
				2	Male	NPN (FRFE)
4	Female	9	1	1	Female	PNP (FEIS)
				2	Female	NPN (ISFE)
5	Female	9	1	1	Female	PNP (FEFR)
				2	Female	NPN (FRFE)
6	Female	4	1	1	Female	PNP (FEIS)
				2	Female	NPN (ISFE)
7	Male	12	1	1	Female	PNP (FEFR)
				2	Female	NPN (FRFE)
8	Male	4	1	1	Female	PNP (FEIS)
				2	Female	NPN (ISFE)
9	Male	9	1	1	Female	PNP (FEFR)
				2	Female	NPN (FRFE)
10	Female	5	1	1	Male	PNP (FEIS)
				2	Male	NPN (ISFE)
11	Female	NA	1	1	Male	PNP (FEFR)
				2	Male	NPN (FRFE)
12	Female	8	1	1	Male	PNP (FEIS)
				2	Male	NPN (ISFE)
13	Male	7	2	1	Male	NPN (FRFE)
				2	Male	PNP (FEFR)
14	Male	9	2	1	Male	NPN (ISFE)
				2	Male	PNP (FEIS)
15	Male	10	2	1	Male	NPN (FRFE)
				2	Male	PNP (FEFR)
16	Female	3	2	1	Female	NPN (ISFE)
				2	Female	PNP (FEIS)
17	Female	3	2	1	Female	NPN (FRFE)
				2	Female	PNP (FEFR)
18	Female	11	2	1	Female	NPN (ISFE)
				2	Female	PNP (FEIS)
19	Male	NA	2	1	Female	NPN (FRFE)
				2	Female	PNP (FEFR)
20	Male	4	2	1	Female	NPN (ISFE)
				2	Female	PNP (FEIS)
21	Male	13	2	1	Female	NPN (FRFE)
				2	Female	PNP (FEFR)
22	Female	NA	2	1	Male	NPN (ISFE)
				2	Male	PNP (FEIS)
23	Female	5	2	1	Male	NPN (FRFE)
				2	Male	PNP (FEFR)
24	Female	12	2	1	Male	NPN (ISFE)
				2	Male	PNP (FEIS)

### Sound recordings

The vocalisations used in this study were obtained from a previous study [7] conducted at the same location. The calls selected belonged to goats that did not share a pen with the subjects during the night, or to goats that were no longer at the sanctuary at the time of testing. Calls were recorded at distances of 3–5 m from the focal animal using a Sennheiser MKH-70 directional microphone (frequency response 50–20,000 Hz; max SPL 124 dB at 1 kHz) connected to a Marantz PMD-660 digital recorder (sampling rate: 44.1 kHz with amplitude resolution of 16 bits in WAV format). Three different contexts inducing emotions were considered: 1) food anticipation (positive, high arousal), in which the goats, tested in pairs in two adjacent pens, learned to anticipate a food reward after three days of training and were recorded on the fourth day when the experimenter approached the tested goats with a bucket of food; 2) food frustration (negative, high arousal), in which only one of the goats in a pair received food from the experimenter, and the other one was recorded while its pair mate was eating; 3) isolation (negative, low arousal), in which the tested goats were recorded while isolated in a pen alone for 5 min away from the other goats but within their usual daytime range, after 3 days of habituation to this situation. The changes in the behaviour and physiology of the subjects in these three contexts were examined. The arousal and the valence of each recording context were determined using physiological and behavioural indicators of emotions (used to validate of the emotional arousal and valence; [7]. Food anticipation and food frustration induced higher arousal compared to isolation. Food anticipation and food frustration were also associated with lower heart-rate variability, higher respiration rate, more movements, more calls, more time spent with ears pointing forwards and less time with ears on the side. In the food anticipation condition, goats had their ears oriented backwards less often and spent more time with their tails up compared to the food frustration and isolation conditions [7]. The detailed vocal parameter analysis identified six acoustic parameters affected by the arousal. F0 contour over time and energy quartile increased with arousal, whereas the first formant decreased. F0 variation within the call was influenced by valence and decreased from negative to positive valence. The acoustic structure of the calls is described in more detail in Briefer et al. [7].

### Playback experiments and exclusion criteria

The habituation-dishabituation-rehabituation paradigm (modified from Charlton et al., [37, 48, 49]) was used to investigate whether goats are able to perceive conspecific vocal expression of emotional valence. The paradigm is based on the repeated presentation of a stimulus, for example a positive call produced while a goat was experiencing a given emotional valence, to a subject (habituation), followed by the presentation of a different stimulus [dishabituation; in our case, calls produced while a goat was experiencing a situation with emotional valence opposite to the situation used during the habituation phase (e.g. negative)]. The response (behavioural and/or physiological) of the subject should indicate whether the element that distinguishes the two stimuli (in our case, change in valence) is conspicuous enough to be detected. A reduction in the response of the subject (habituation) after a repeated presentation of the stimulus, followed by an increment in the response when a new stimulus is presented (dishabituation) would indicate that the two stimuli are perceived as different [48, 49, 71]. After the dishabituation, the stimulus used in the habituation is presented again (rehabituation), in order to ensure that the response occurring during the dishabituation is robust and not a random consequence of a renewal of attention [48, 49].

Twenty four sessions (six goats in total, playback sequences played FEFR = 5, FRFE = 7, FEIS = 8, ISFE = 4) were excluded from the final analysis because: 1) subjects did not react to the first habituation call, i.e. individuals did not look towards the source of the playback during the first call of habituation, and/or 2) subjects failed to habituate, defined as sessions where the time spent looking towards the speaker during the last playback of the habituation phase was more than two times longer than the first playback of the habituation phase [37].

### Playback sequence and procedure

Each playback sequence consisted of 13 calls, separated by a time interval of 20 s. Only good quality calls with low background noise were selected to prepare the playback sequences as follows: three calls per individual with a signal-to-noise ratio > 10 dB were selected from eight individuals in the food anticipation context, from six individuals in the food frustration context and from five individuals in the isolation context (i.e. 57 calls in total) within the original pool of 180 calls (i.e. 40 calls in food anticipation; 80 calls in food frustration and 60 calls in isolation; [7]. In order to test if the valence of the calls was perceived regardless of context (two contexts of negative valence; frustration and isolation) and order (i.e. which valence was used for the habituation or dishabituation phase), the sequences included the following combinations of valence and context: six sequences included food anticipation (habituation) – food frustration (dishabituation) – food anticipation (rehabituation) calls, (hereafter, “FEFR”); six sequences included food frustration (habituation) – food anticipation (dishabituation) – food frustration (rehabituation) calls, (hereafter, “FRFE”); five sequences included food anticipation (habituation) - isolation (dishabituation) – food anticipation (rehabituation) calls, (hereafter, “FEIS”); and five sequences included isolation (habituation) – food anticipation (dishabituation) - isolation (rehabituation) calls, (hereafter, “ISFE”).

Calls within the sequence were emitted by the same individual, but were produced in two different emotional contexts. The first nine calls (three different calls produced in a given context – food anticipation, food frustration or isolation - repeated three times each and combined in random order) constituted the habituation phase (H); the following three calls (three different calls produced in a context of opposite valence compared to the habituation calls, and combined in a random order) constituted the dishabituation phase (D); and the final call (a single call randomly selected from the habituation phase) constituted the rehabituation phase (R).

Each vocalisation was broadcasted from a Mackie Thump TH-12A loudspeaker (LOUD Technologies Inc., Woodinville, WA; frequency response: 57 Hz - 20 kHz ± 3 dB) connected to an active box to boost the sound (Active Box DI-100 Fame) and to an audio player (Technika MP111), at an approximately natural amplitude (88.99 ± 0.93 dB) measured at 1 m using an ASL-8851 sound level meter. The original duration of the calls was maintained, in order not to remove any information contained in their structure (feeding = 0.71 ± 0.02 s; frustration = 0.70 ± 0.03 s and isolation = 0.71 ± 0.02 s). The peak amplitude of each call had been equalised during the preparation of the sequences. The presentation order of the playback sequences was balanced within each group of 12 subjects (tested in the same day), so that half of the subjects experienced first the Positive – Negative - Positive (PNP) sequence and the opposite Negative – Positive - Negative (NPN) sequence in the following session. The other half of the group experienced NPN first and PNP in the following session. The sex of the goat that produced the calls used in the playback sequence was counterbalanced within and between subjects (half the males and half the females were tested with same sex playbacks and the other half with opposite sex playback). Overall, each subject was tested on two different days, with one session per day, and a three-day interval between sessions.

Before the experiment started, goats were released twice (i.e. one for each consecutive day) for 5 min inside the arena to familiarise with the experimental setup. During the test phase, individuals were gently brought to the start pen, where a familiar experimenter placed the heart rate monitor BioHarness belt around the goats’ thorax. When a clear electrocardiogram (ECG) was obtained, the main gate that provided access to the central arena was opened. After 30 s, the first playback call was played and the session continued until the last call was played.

### Behavioural and physiological data collection and analyses

The duration of looking towards the speaker was measured and defined as the time from when the subject directed the head towards the playback location (start) until when the head was turned away and the animal stopped looking (end), within the 20 s following each call. If the subjects were already looking towards the speaker when one of the calls of a sequence was broadcasted, then this behaviour was considered to begin at the onset of the playback [48]. When the goat looked away and then looked back to the speaker within the 20 s following each call, the time was scored again. The total duration of looking towards the sound source was calculated for each subject and for each of the 13 calls. All trials were video recorded using a digital video camera placed at the entrance of the arena (Sony HDR-CX190E). The videos were analysed frame by frame using QuickTime player (Apple Inc.). A second observer, blind to the experimental hypothesis, scored 30% of the sessions to test the reliability of the parameters measured by the two observers. Inter-observer agreement for the behaviour scored was high (Spearman rank correlation; *r*_*s*_ = 0.990, *p* < 0.001).

The physiological parameters were recorded using a non-invasive Bluetooth device (EC38 Type 3, BioHarness Physiology Monitoring System, Zephyr Technology Corporation, Annapolis, MD, USA) fixed to a belt placed around the goat’s chest. A small patch of hair (7 cm X 15 cm) was clipped before the experiment in order to obtain a clearer ECG trace. This procedure took place a week before the testing to avoid any confounding effects of being manipulated. The continuous ECG trace was transmitted in real time to a laptop (ASUS S200E) and registered using the software AcqKnowledge v.4.4 (BIOPAC System Inc.). During the playbacks, we entered visible markers in the ECG trace at the beginning of each call to be able to link the physiological data to the specific calls and phases of the experiments. The time of occurrence of each heart beat identified on the ECG trace was extracted during the 20 s following each call. HR and HRV (measured as root mean square of successive inter-beat interval differences, RMSSD) were further calculated from the extracted heart beats on the longest selection possible within 20 s.

### Data analysis

Analyses were conducted using Linear and Generalised Mixed-Effects Models (lmer function, lme4 library; Pinheiro 2000) in R v.3.2.2 [72, 73]. First, the occurrence of looking towards the speaker, HR and RMSSD were compared over the nine calls played during the habituation phase (H1-H9) to determine whether goats habituated to the sounds throughout this phase (indicated by a significant decrease in occurrence of looking and in HR throughout the phase). Subsequently, responses to the last habituation call (H9) were compared to those of the first dishabituation call (D10). Responses were also compared to dishabituation calls D10 vs D11, and D11 vs D12, to investigate the response pattern within the dishabituation phase. Finally, responses to the dishabituation calls (D10, D11, and D12) were compared to those of the rehabituation call (R13). The model selection and the variable considered were call number (1 to 13; or a combination of these for further post-hoc tests) and call valence (positive or negative), as well as their interaction as fixed effects. The duration of the measurement period (9.34 ± 0.17 s) was also included as a control factor in the model carried out on RMSSD, because it could potentially affect this value. The factor “Session” [1 and 2] nested within the identity of the goats (“ID”) nested within “Group” [1 and 2] was included as a random factor, crossed with the identity and the sex of the goat producing the playback calls. Non-significant interactions between call number and valence were removed from the models [74]. The statistical significance of the factors was assessed by comparing the models with and without the factor included using a likelihood-ratio test. When an interaction effect was found, further post-hoc comparisons were performed using a Tukey HSD test.

Q–Q plots and scatterplots of the residuals of the model were checked visually for normal distribution and homoscedasticity. In order to meet the model assumptions, HR was log-transformed. HR (log-transformed) and RMSSD were input into LMMs fit with Gaussian family distribution and identity link function. The occurrence of looking towards the speaker did not meet the assumptions despite log-transformation. It was thus transformed to binary data (looked at the speaker = 1; did not look = 0) and input into a GLMM fit with binomial family distribution and logit link function.

## Data Availability

All data generated or analysed during the study are included in this submitted article and its supplementary information file.
